# The Role of the Flagellar Protein FlgJ in the Virulence of *Brucella abortus*

**DOI:** 10.3389/fcimb.2020.00178

**Published:** 2020-04-28

**Authors:** Roberto F. Coloma-Rivero, Leonardo Gómez, Francisco Alvarez, Waleska Saitz, Felipe del Canto, Sandra Céspedes, Roberto Vidal, Angel A. Oñate

**Affiliations:** ^1^Laboratory of Molecular Immunology, Department of Microbiology, Faculty of Biological Sciences, Universidad de Concepción, Concepción, Chile; ^2^Microbiology and Mycology Program, Institute of Biomedical Sciences, Faculty of Medicine, University of Chile, Santiago, Chile

**Keywords:** *Brucella abortus*, genomic island 3 (GI-3), flagellum, FlgJ protein, intracellular trafficking, virulence factors

## Abstract

*Brucella abortus* is a facultative intracellular pathogen that causes a zoonosis called brucellosis. This disease leads to abortion and infertility in cattle, and diverse complications in humans. *B. abortus* is a successful intracellular bacterium that has developed the ability to evade the host's immune system and it replicates in professional and non-professional phagocytic cells, persisting in the different tissues, and organs of its hosts. It has been described that *Brucella* expresses a polar flagellum under certain conditions, but its function is still unknown. In this study we evaluated the role of the FlgJ, a protein, presumably a peptidoglycan hydrolase involved in flagellum formation and in the virulence of *B. abortus* strain 2308. *B. abortus* 2308 Δ*flgJ* mutant and complemented strains were constructed to study the function of the FlgJ protein in the context of the virulence of this pathogen in *in vitro* and *in vivo* assays. The results showed that the elimination of the *flgJ* gene delays the growth rate of *B. abortus* in culture, reduces its intracellular survival capacity in professional and non-professional phagocytic cells, rendering it unable to escape from the endocytic route and not reaching the endoplasmic reticulum. It also negatively affects their persistence in BALB/c mice. Functionally, the *B. abortus* 2308 *flgJ* gene restored motility to an *E. coli flgJ* mutant gene. Furthermore, it was discovered that the production of FlgJ protein is associated with the bacterial adherence by *B. abortus*. Therefore, although the specific function of the polar flagellum for *Brucella* is unknown, the data indicates that the flagellar *flgJ* gene and its product are required for full virulence of *B. abortus* 2308, since its deletion significantly reduces the fitness of this pathogen *in vitro* and *in vivo*.

## Introduction

*Brucella abortus* is a Gram-negative bacterium that causes of bovine brucellosis, a global zoonotic disease affecting cattle and humans (Corbel, 1997). This pathogen can infect humans through direct contact with infected animals, the ingestion of contaminated food or by the inhalation of aerosolized particles contaminated with *B. abortus* (De Figueiredo et al., 2015). In the mucosal membranes, this bacterium can be captured by phagocytic cells, where it survives to the intracellular microbicide mechanisms due to several virulence factors such as its atypical lipopolysaccharide (LPS), β1,2-glucans, the two-component system BvrR/BvrS, or the type 4 secretion system virB (Carvalho et al., 2010). These virulence factors allow it to inhibit the phagosome-lysosome fusion and to escape toward the endoplasmic reticulum to develop its replicative niche (Zygmunt et al., 2012; Altamirano-Silva et al., 2018). This capacity for intracellularly survival in phagocytic cells is fundamental to reaching several tissues and organs, producing a systemic infection, which in humans present as undulant fever, night sweats, insomnia and headache, followed by a chronic phase where this bacterium is localized in various tissues and organs causing hepatitis, neurobrucellosis, or endocarditis (Galinska and Zagórski, 2013; Dean et al., 2014; Young et al., 2014). In cattle, brucellosis produces mainly abortion and infertility in females and males, respectively (D'Anastasio et al., 2011).

*B. abortus* is a bacterium traditionally described as non-motile; however, it has all the flagellar genes for synthesis of a functional flagellum contained in its genome (Abdallah et al., 2003). This flagellum was reported in *Brucella melitensis* 16M as a polar and sheathed apparatus, which is expressed under precise *in vitro* conditions and during infection (Fretin et al., 2005). Mutant strains for flagellar proteins such as *fliF, flhA, motB*, or *flgE* were shown to be required for the intracellularly survival of *B. melitensis* in mouse spleen (Fretin et al., 2005). The bacterial flagellum has commonly been associated with several functions that differ between bacteria or the bacterial life cycle: a scourge can, e.g., participate in biofilm formation or adherence (Haiko and Westerlund-Wikström, 2013). Several bacteria colonize different surfaces and invade susceptible hosts causing chronic infections that grow predominantly as biofilms (Hall-Stoodleyl and Stoodley, 2009; Burmølle et al., 2010). The biofilms are extracellular polymeric substances (EPS) self-produced by microorganisms, which are mainly polysaccharides, proteins, nucleic acids and lipids that mediate their adhesion to diverse surfaces and allow intense interactions among bacteria (cell-cell communication, competition, cooperation or horizontal gene transfer) (Flemming and Wingender, 2010).

The underlying molecular mechanisms of *B. abortus* flagellum or biofilm formation has been poorly studied. However, it has been demonstrated that quorum-sensing (QS) genes, vjbR and blxR, transcriptional regulator is involved in Brucella virulence (Rambow-Larsen et al., 2008). One of these, VjbR, is required by *B. melitensis* for the transcription of the type IV secretion system and expression of various flagellar genes (*fliF, flhA, motB*, or *flgE*), which contribute its virulence in mice (Delrue et al., 2005; Fretin et al., 2005). Interestingly, *B. abortus* contains the flagellar protein FlgJ encoded out of a flagellar gene cluster, specifically in the open reading frame (ORF) BAB1_0260 of the *B. abortus* genomic island 3 (GI-3), a GI constituted by several ORFs, some of them involved in survival, replication and immune evasion (Rajashekara et al., 2004; Cirl et al., 2008; Salcedo et al., 2008; Céspedes et al., 2012; Ortiz-Román et al., 2014; Gómez et al., 2016, 2018). This protein is and ortholog to FlgJ of *E. coli* and *Salmonella* enterica serovar Typhimurium, and it plays an important role in the flagellum assembly. FlgJ is characterized by a N-terminal half with function scaffold or cap essential for flagellar rod assembly and a C-terminal half with peptidoglycan (PG) hydrolyzing activity that facilitates rod penetration into the PG (Nambu et al., 1999; Hirano et al., 2001). In *E. coli* K-12 strain, this peptidoglycan hydrolase FlgJ has muramidase activity by glucosaminidase or Lysozyme subfamily 2 (LYZ2) domains which hydrolyses the peptidoglycan layer and assembly the rod structure in the periplasmic space (Marchler-Bauer et al., 2017). Their function hydrolyzing is generated by glucosaminidase domains of the Carbohydrate Active Enzyme (CAZy) family GH73 facilitating the passage of the flagellum by the cleaving of the β-1,4 glycosidic bond between β-N-acetylglucosamine and β-N-acetylmuramic acid sugars comprising the glycan strands of the PG (Hirano et al., 2001; Zaloba et al., 2016).

Although the importance of the FlgJ protein in the physiology or pathogenicity of *Brucella* has not been described, it could potentially be involved in virulence because the vaccination of mice with recombinant FlgJ protein conferred significant protection levels against infection with *B. abortus* strain 544 (Li et al., 2012). In general, the process of bacterial infection includes adhesion, invasion, escape of infected cells and modulation of the immune response that contributes to persistence inside the host, this kind of interactions with the hosts is linked to the function expression flagella in pathogenic and symbiotic bacteria (Josenhans and Suerbaum, 2002). The expression of *Brucella* flagellar proteins could play a role in these infection processes or act directly in the growth and division of this bacterium, and thus fulfill important roles in the physiology and interaction with their host, where these FlgJ proteins are considered important part of this process. Therefore, in the present study, the role of the flagellar protein FlgJ in the virulence of *B. abortus* strain 2308 was investigated. Using an isogenic *B. abortus* 2308 Δ*flgJ* mutant it was determined that the FlgJ protein is required for flagella function, and that this flagellum is required for full virulence in phagocytic cells and in persistence in mice.

## Materials and Methods

### Animals

Ten-week old female isogenic BALB/c mice were obtained from the Instituto de Salud Pública (Santiago, Chile). The animals were kept at the Laboratory of Molecular Immunology (Department of Microbiology, Faculty of Biological Sciences, Universidad de Concepción, Chile) and after arrival were randomly distributed into experimental and control groups and allowed to acclimate. The mice were kept in a under controlled temperature and fed with commercial pellets and water *ad libitum*. All regulations from the Institutional Bioethics Committee of the Faculty of Biological Sciences, Universidad de Concepción, were fulfilled. The Bioethics and Safety Committee of the Faculty of Biological Sciences at the Universidad de Concepción approved this study. All efforts were made to minimize animal suffering.

### Cell Lines

In this work, HeLa cells and RAW and J774.A1 macrophage cell lines obtained from the American Type Culture Collection (ATCC) were used. The cells were cultured in Dulbecco's Modified Eagle Medium (DMEM) (ThermoFisher Scientific, MA) and supplemented with 10% fetal bovine serum (Gibco BRL, USA) and antibiotic-antimycotic solution (100 IU of penicillin, 100 μg/mL of streptomycin, and 0.25 μg/mL of amphotericin, Sigma-Aldich Co., MO).

### Bacterial Strains and Culture Conditions

The bacterial strains used in this study are listed in Table 1. *Escherichia coli* and *B. abortus* strains were cultured in Luria Bertani (LB), Terrific broth or Brucella broth (Becton, Dickinson and Company, BD, Sparks, MD21152 USA), respectively. All *B. abortus* strains were cultured for 48–72 h at 37°C under microaerophilic conditions and supplemented with antibiotics. Furthermore, *E. coli* strains were cultured in Terrific broth medium (BioWorld, Ohio, USA) or agar for 24 h at 37°C. When was necessary, the bacterial medium was supplemented with 50 μg/ml of kanamycin, 100 μg/ml of ampicillin or 30 μg/ml of chloramphenicol (Ortiz-Román et al., 2014).

**Table 1:** Bacteria and plasmids used in this study.

**Strains or plasmids**	**Characteristics**	**Reference**
*Brucella abortus* 2308	Wild-type, smooth, virulent strain	Laboratory stock
*B. abortus* 2308 Δ*flgJ*	*B. abortus* 2308, deleted in the BAB1_0260 ORF (*flgJ*)	This work
*B. abortus* 2308 Δ*flgJ* (pVB1-*flgJ*)	*B. abortus* Δ*flgJ*, containing plasmid pVB1-*flgJ*, Am^r^, Km^r^	This work
*B. abortus*2308-*gfp*	Wild-type strain, containing plasmid pAK*gfp*1, Amp^r^	This work
*B. abortus* 2308 Δ*flgJ-gfp*	*B. abortus* Δ*flgJ* containing plasmid pAK*gfp*1, Amp^r^, Km^r^	This work
*B. abortus* 2308 Δ*flgJ* (pVB1-*flgJ*)-*gfp*	*B. abortus* Δ*flgJ* (pVB1-*flgJ*) containing plasmid pAK*gfp*1, Am^r^, Km^r^	This work
*Escherichia coli* DH5α	*F– Φ80lacZΔM15 Δ(lacZYA-argF) U169 recA1 endA1 hsdR17* multiplicity of plasmids	Invitrogen
*E. coli* K-12	*(rK–, mK+) phoA supE44 λ- thi-1 gyrA96 relA1*	Invitrogen
*E. coli* Δ*flgJ*	*E. coli* deleted in the *flgJ* gene	This work
*E. coli* Δ*flgJ* (pVB1-*flgJ*)	*E. coli* Δ*flgJ* complemented with vector pVB1-*flgJ*	This work
pSIM7/pSIM9	Broad-host-range cloning vector, Lambda Red Recombinase (λ-Red)	Laboratory stock
pKD4	Km^r^ sequence	Laboratory stock
pVB1	Cloning vector for PCR product expression	Laboratory stock
pVB1-*flgJ*	Recombinant vector codifying of *B. abortus* 2308*flgJ* gene	This work
pAK*gfp*1	Plasmid codifying of green fluorescence protein (GFP) (Amp^r^)	Addgene

*Am^r^ - Ampicillin resistance; Km^r^ - Kanamycin resistance*.

### Construction of *flgJ* Mutants in *B. abortus* and *E. coli* K12

In order to generate a *B. abortus* Δ*flgJ* mutant, a modification of the phage lambda (λ) red system we used (Datsenko and Wanner, 2000). For this, 10^10^ CFU mL^−1^ of *B. abortus* 2308 were transformed by electroporation with 200 ng of recombinant plasmid pSIM7 (Sharan et al., 2009) and incubated at 30°C for 72 h in brucella broth supplemented with 30 μg/mL of chloramphenicol. Transformants were incubated at 42°C for 30 min to induce expression of the λ red recombinase (Halling, 1998) and to allow the kanamycin resistance cassette (Km^r^), previously amplified by PCR from plasmid pKD4 (Table 2) to exchange by homologous recombination. The PCR product was purified and electroporated in 10^10^ CFU/mL of *B. abortus* 2308 previously transformed with pSIM7 and cultured at 37°C for 72 h in brucella agar plates supplemented with 50 μg/mL Km for selection of mutants. Colonies were screened by PCR using primers *flgJ* km^r^ (FW) and *flgJ* km^r^ (RV) (Table 2). In order to ensure that the possible changes observed were only in the mutant strain Δ*flgJ*, this strain was supplemented with the respective gene linked to the vector pVB1. Briefly, *flgJ* gene was amplified from *B. abortus* 2308 genomic DNA using primers that carry cut sequences for the restriction enzymes NdeI and BamHI at their ends (Table 2). Then, the amplified fragment was purified and cloned into pVB1 using the enzyme T4 DNA ligase to generate the pVB1-*flgJ* construct. The plasmid pVB1-*flgJ* was electroporated into *B. abortus* 2308 ΔflgJ, generating the strain complemented with *B. abortus* 2308 Δ*flgJ* (pVB1-*flgJ*) (Ortiz-Román et al., 2014). It should be mentioned that the *E. coli* used in this work was mutated using the same protocol described previously.

**Table 2:** Primers used in this study within the genome framework of *B. abortus* 2308.

**Name**	**Sequence**	**Restriction enzyme**	**Size(pb)**
*flgJ* Km^r^ (F)	TATATCTGATCCGGGTTTTCACCGAAGAAAAGCAAGCCTTGAAAGAGCAGGCCCGCAAGAAAGGTACTTCGCTT TCCGGCTTGATCCGGGATGCTGTTCTTGATGCGCCTTC AACTTCGACAGCGGGATACGATGGAGTGTGGTCATGAGTGTAGGCTGGAGCTGCTTC^*^	NA	
*flgJ* Km^r^ (R)	AACGTCTTTTCGCCAATTCGTCGGGCCTACTGTACCGTCATTCGAGAATGACTGATTGGCAAACCGCTTTTGCAA TGCCGACCGCATTACGTCTGGAGACGTGCCCTCTGGG AATTCGGCTATCGTGCCGTCTGGAAGCTCTACTTCAATCATATGAATATCCTCCTTAG^*^	NA	
*flgJ* (F)	AAGAAAAGCAAGCCTTGAAAGAG		
*flgJ* (R)	CATGACCACCTCCATCCATCGTATC		
*flgJ*	ACTGA*CATATG*ACACCTATCGGCAACAGAAAT	*NdeI*^†^	2118
*flgJ*	AG*GGATCC*TCATTCGAAATCACCAGTCTGC	*Bam*HI^†^	

* *The Sequence used to amplify Km^r^ is underlined*.

† *NdeI and BamHI, restriction endonuclease cleavage sites are underlined*.

*NA, non-availability; (F), Forward; (R), Reverse*.

### Growth Curves

To determine whether the mutation of the *flgJ* gene affected the growth rate of *B. abortus* 2308, growth curves for *B. abortus* 2308Δ*flgJ* and *B. abortus* Δ*flgJ* (pVB1-*flgJ*) were performed and compared to wild type *B. abortus* 2308. To this end, 100 μl of each strain at an optical density at 600 nm (OD_600_) of 0.5 were added into flasks with brucella broth and grown with agitation (150 rpm) at 37°C. Aliquots of these cultures were taken every 12 h for a period of 144 h and their OD_600_ was measured.

### Transcomplementation and Motility Assays

To demonstrate the role of the *B. abortus flgJ* gene in flagellar function, a mutant *E. coli* strain K-12 for the *flgJ* gene (*E. coli* Δ*flgJ*) was constructed, which was complemented with the *B. abortus* 2308 *flgJ* gene, yielding *E. coli* Δ*flgJ* (pVB1-*flgJ*). The function of this flagellar protein in the motility of the constructs was evaluated using Motility-Indole-Ornithine (MIO) medium (Becton Dickinson) for 24 h at 37° C.

### Screening for Biomass Production

To assess the participation of the FlgJ protein in the formation of adherent biomass, 100 μl (1.5 × 10^8^ CFU ml^−1^) of *Brucella abortus* 2308, *Brucella abortus* 2308 Δ*flgJ* and the *Brucella abortus* Δ*flgJ* (pVB1-*flgJ*) were each grown independently in brucella broth, using *E. coli* strain K-12 (Almirón et al., 2013) as an external control. These strains were added to 96-well microplates. The plates were incubated for 7 days at 37°C without shaking them, and non-adherent bacteria were removed by washing three times with sterile physiological saline solution (0.9% NaCl, w/v). The adherent bacteria (biomass adhered) were then stained for 45 min with a solution of 1% Crystal Violet (Sigma-Aldrich, w/v). After washing and air drying, the stain bound to the adherent cells was dissolved into ethanol and the adherent biomass, per each well, was measured as the optical density (OD 550 nm) as an indirect index of adherent biomass formation, using a Multiskan GO Microplate Spectrophotometer (Thermo Scientific Lab.) (Stepanović et al., 2007; Spanò et al., 2016; Chai et al., 2017). All the experiments in this study were performed three times; the supernatant was not measured and it was not verified that the cell bodies present in it had any growth activity.

### Intracellular Survival of *B. abortus* Strains

Intracellular replication of *B. abortus* 2308, *B. abortus* 2308 Δ*flgJ* and *B. abortus* 2308 Δ*flgJ* (pBV1-*flgJ*) was evaluated in non-professional (epithelial HeLa) and professional (J774.A1 macrophages) phagocytic cell lines culture in supplemented DMEM medium in a 5% CO_2_ atmosphere at 37°C. In parallel, all *B. abortus* strains were cultured in brucella broth for 48 h and suspended at a concentration of 10^7^ CFU ml^−1^ of DMEM (supplemented with 10% fetal calf serum and 2 mM glutamine, free of antibiotics). These bacterial suspensions were added to HeLa or J774.A1 cells at a 500:1 or 50:1 multiplicity of infection (MOI), respectively. After 1 h of incubation, the cell monolayer was washed with phosphate-buffered saline (PBS) and incubated for 60 min with fresh media, supplemented with 50 μg mL^−1^ gentamicin and 100 μg mL^−1^ streptomycin for extracellular bacteria elimination. At 4, 24, 48, and 72 h post-infection, the cells were washed with PBS and lysed with 1 mL of 0.1% Triton X-100. The cellular lysate was serially diluted and the number of CFU mL^−1^ was determined in brucella agar plates (Céspedes et al., 2011, 2012).

### Intracellular Trafficking of *B. abortus* 2308 Δ*flgJ* in Macrophages

To visualize the intracellular trafficking of *B. abortus* strains by means of immunofluorescence techniques, they were transformed with the host-wide vector pAKgfp1 (Addgene plasmid #16076) encoding the green fluorescent protein (GFP). Intracellular trafficking of the *Brucella* strains within RAW264.7 murine macrophages (MOI 50:1) were quantified in early and late endosomes and reticulum endoplasmic at 15 minutes, 4 and 12 h post infection (pi), respectively. Next, macrophages were fixed with 4% paraformaldehyde, washed with PBS (pH 7.4) and incubated with goat anti-EEA1 polyclonal antibodies (Santa Cruz Biotechnology, Dallas, TX, USA), specific for an early endosome marker (Early Endosome Antigen 1), rabbit anti-LAMP1 (lysosomal-associated membrane protein 1), a late endosomal marker (Santa Cruz Biotechnology, Dallas, TX, USA) and goat anti-calnexin for endoplasmic reticulum (ER) marker (Abcam, Cambridge, United Kingdom). All antibodies were diluted in PBS buffer (pH 7.4) with 0.5% bovine serum albumin (BSA) and incubated in a humidity chamber for 3 h. After this period, coverslips were washed with PBS (pH 7.4) and incubated with donkey anti-goat IgG Alexa Fluor 594 (Thermo Fisher Scientific Inc., Massachusetts, MA, USA) or donkey anti-rabbit IgG Alexa Fluor 647 as a secondary antibody (Abcam, Cambridge, UK) diluted 1:500. Finally, the samples were mounted on slides using Dako Cytomation fluorescent mounting medium (Sigma-Aldrich, St. Louis, MO, USA). The samples were observed under a Zeiss LSM 700 laser scanning confocal microscope for image acquisition (Zeiss, Oberkochen, Germany). The intracellular co-localization data and images of 1,024 X 1,024 pixels were acquired and assembled by image analysis using ImageJ software. Data are representative of at least two independent experiments.

### Bacterial Colonization Assay in Spleen of BALB /c Mice

*B. abortus* wild-type 2308, mutant and complemented strains were used to infect mice, and the survival of bacteria in the spleen was determined (Fretin et al., 2005). Nine-week-old, pathogen-free, female BALB/c mice, the most extensively used model for studying chronic infection caused by *Brucella* spp. (Blocker et al., 2003), were inoculated intraperitoneally with 10^5^ CFU^−1^ of each *Brucella* strain in 0.1 ml of PBS. Two - and four -week post-infection, animals were euthanized by cervical dislocation, and their spleens were removed and homogenized in PBS. The homogenized tissues were serially diluted in PBS and plated onto Columbia agar with 5% sheep blood (Biomerieux, USA to determine the number of CFUs per spleen (Ortiz-Román et al., 2014).

### Statistical Analysis

Data analysis to establish significant differences in adherent biomass formation and intracellular trafficking between *B. abortus* 2308 Δ*flgJ, B. abortus* 2308 Δ*flgJ* (pVB1-*flgJ*) and *B. abortus* 2308 strains were analyzed by one-way ANOVA, while the intracellular survival in professional and non-professional phagocytic cells and mice infection assays were analyzed using a two-way ANOVA. Tukey's multiple comparison test analyzed all results. Values of *P* < 0.05 were considered statistically significant. All the quantitative experiments were performed three times on separate days in triplicate and the results shown are the mean of those experiments.

## Results

### Growth Curve of Different *B. abortus* Strains

**Figure 1:**
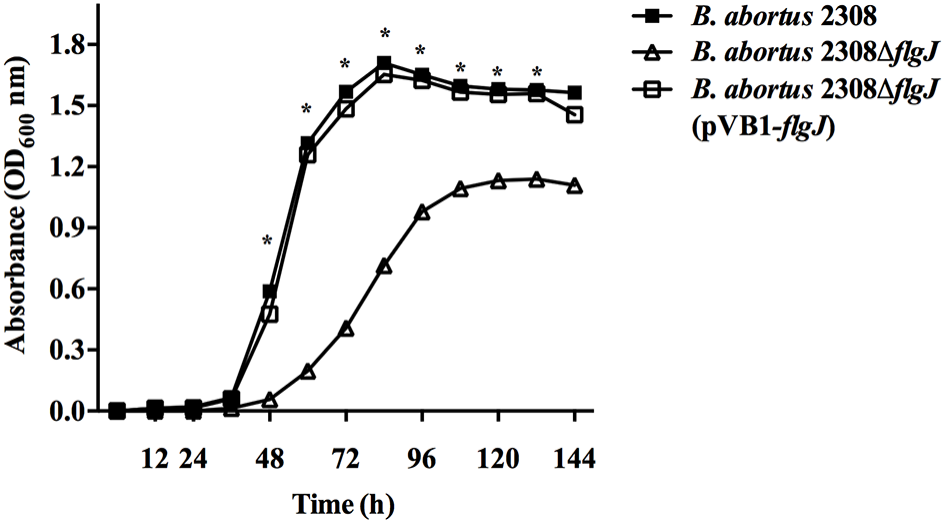
Growth curves of *B. abortus* strains; *B. abortus* 2308 (wt), *B. abortus* 2308Δ*flgJ*, and complemented 2308Δ*flgJ* (pBV1-*flgJ*). Deletion of flgJ gen in *B. abortus* 2308 results in a growth deficiency. The absorbance (optical density, OD) was measured under 600 nm; the plotted points for each curve was measured at 12 h of culture. Results were expressed as mean ± standard deviation (SD), *P < 0.05 significant.

The effect of the mutation of the *flgJ* gene on bacterial growth was evaluated as described above. The *B. abortus* 2308 Δ*flgJ* showed a 12 h-delay in the lag phase as compared to the wild-type strain. In addition, when observing the logarithmic growth phase of the mutant strain, it was much lower than the wt strain. *B. abortus* 2308 Δ*flgJ* reached the stationary phase after 96 h of culture; by contrast, the wild-type strain entered the stationary phase after 72 h of growth. No differences were observed between the growth curves of *B. abortus* 2308 and *B. abortus* 2308 Δ*flgJ* (pBV1-*flgJ*) (Figure 1). This indicates that the FlgJ protein encoding for ORF BAB1_0260 is involved in the normal growth of *Brucella*.

### Functional Evaluation of the *B. abortus flgJ* Gene

**Figure 2:**
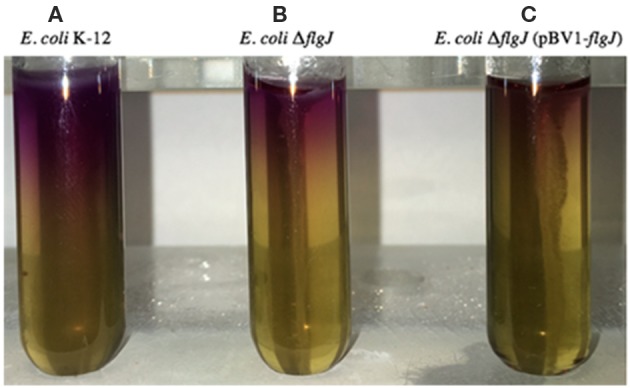
Mobility assay of *E. coli* Δ*flgJ* measured using MIO medium. The lack of the *flgJ* gene evidences a decrease in the mobility of *E. coli* K-12. **(A)** *E. coli* K-12 strain. **(B)** *E. coli* Δ*flgJ strain*. **(C)** *E. coli* Δ*flgJ complemented with* pBV1-*flgJ* encoding of *flgJ* from *B. abortus* 2,308 strain.

The role of the *flgJ* gene in *E. coli* strain K-12 motility was evaluated using an *E. coli* Δ*flgJ* complemented with the *B. abortus flgJ* gene, after inoculation of MIO medium. As expected, the *E. coli* Δ*flgJ* (Figure 2B) lost its motility compared to *E. coli* K12 parental strain (Figure 2A). This defect was slightly rescued by transcomplementation of the *flgJ* gene of *B. abortus* 2308 (Figure 2C).

### Screening for Biomass Production

**Figure 3:**
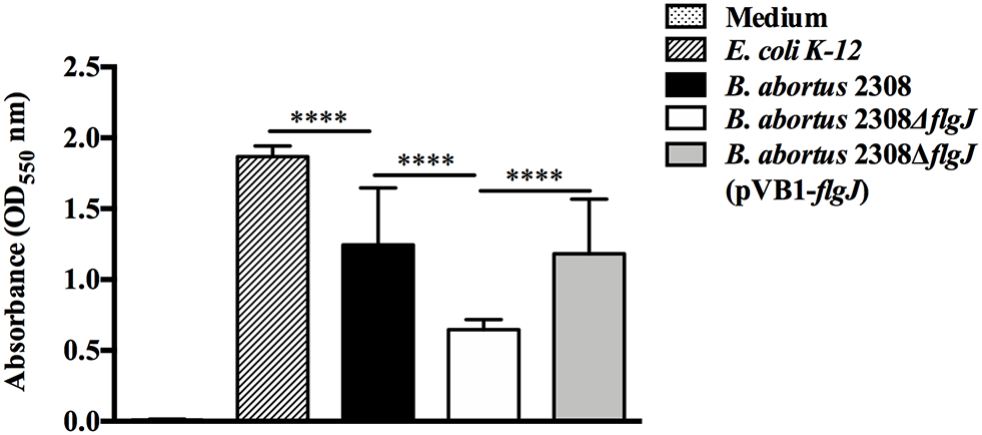
Screening for biomass production. Quantitative measurement by absorbance to 550 nm of adhered biomass production in 96-well polystyrene plates, of *B. abortus* 2,308, *B. abortus* 2308Δ*flgJ*, and complemented 2308Δ*flgJ* (pBV1-*flgJ*) in addition to *E. coli* K-12 as a positive control of biomass production and culture medium as a negative control. Results are expressed as means ± standard deviation. Values of ****P < 0.001 are considered highly significant.

Regarding the ability of the *B. abortus* 2308 strain to form biomass adhered in polystyrene plates at 7 days, the results showed that *B. abortus* 2308 Δ*flgJ* presented a biomass production measured by absorbance at OD_550_ significantly lower than biomass produced by *B. abortus* 2308 Δ*flgJ* (pVB1-*flgJ*) and *B. abortus* 2308 (*P* < 0.001) (Figure 3). These data suggest that deletion of BAB1_0260 from *B. abortus* 2308 reduces its ability to form adhered biomass; therefore, the FlgJ protein could be an actively participant in the formation of biomass adhered on an inert surface.

### Intracellular Survival of *B. abortus* 2308 Δ*flgJ* in Non-professional and Professional Phagocytic Cells

**Figure 4:**
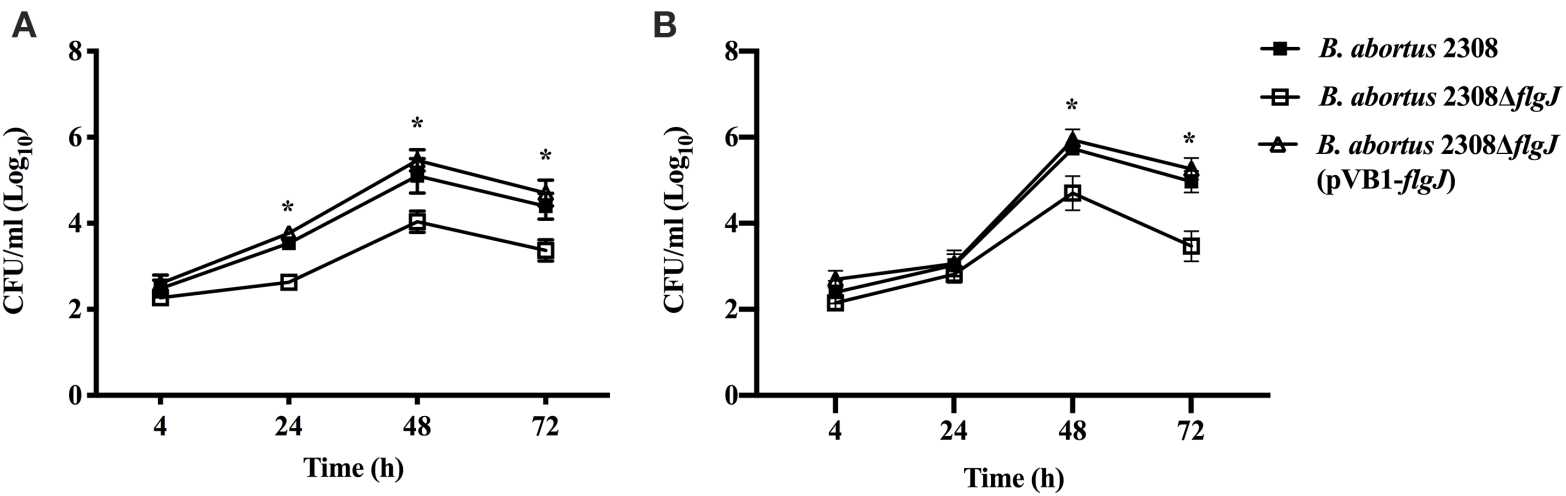
Intracellular survival of *B. abortus* Δ*flgJ* in professional and non-professional phagocytes. **(A)** J774.A1 macrophages were infected at MOI of 50:1. *B. abortus* 2308 (open triangle), *B. abortus* Δ*flgJ* (open square), and complemented *B. abortus* Δ*flgJ* (pBV1-*flgJ*) (closed square). **(B)** HeLa cells were infected at MOI of 500:1. Results correspond to a representative experiment from two separate experiments. For each time point and each strain results are expressed as means ± standard deviation. *P < 0.05 when compared to parental *B. abortus* 2308.

The effect of the deletion of BAB1_0260 ORF (*flgJ* gene) on the ability of *B. abortus* to infect and proliferate within phagocytic and non-phagocytic cells was studied using J774.1 macrophages and HeLa epithelial cells, respectively. We found that at 4 h post-infection (p.i.) of macrophage J774.1, all the *Brucella* strains showed a similar number of intracellular bacteria (Figure 4A). However, between 24 and 72 h p.i., the *B. abortus* 2308 Δ*flgJ* strain was recovered from macrophages approximately in 2 log_10_ CFU ml^−1^ less than *B. abortus* 2308 or *B. abortus* Δ*flgJ* (pVB1-*flgJ*) (*P* < 0.05). When bacterial internalization and survival within HeLa cells was analyzed, all the strains showed a similar number of intracellular bacteria between 4 h (2.3 log_10_ CFU ml^−1^) and 24 h (2.9 log_10_ CFU ml^−1^) p.i. (Figure 4B). However, between 48 and 72 h p.i. the *B. abortus* Δ*flgJ* mutant was recovered at approximately 2.2 log_10_ CFU ml^−1^lower than the parental and complemented strains (*P* > 0.05) (Figure 4). These results indicate that the absence of the *flgJ* gene in *B. abortus* makes it more sensitive to its intracellular survival.

### Effect of the Deletion of the Gene *flgJ* in Intracellular Traffic

**Figure 5:**
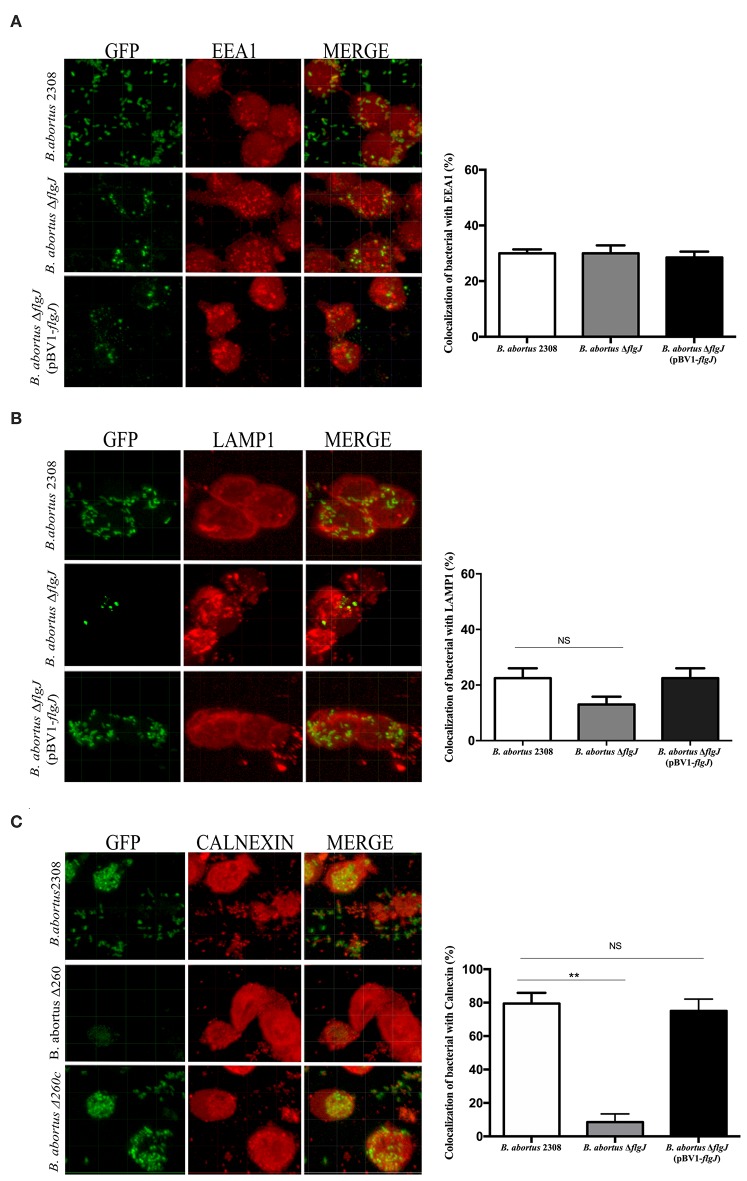
Intracellular trafficking of *B. abortus* 2308 Δ*flgJ* in macrophages. Murine macrophages RAW264.7 were infected with the *Brucella* strains at MOI of 50:1. Representative confocal image showing the co-localization of *B. abortus* 2308-*gfp, B. abortus* Δ*flgJ-gfp* and *B. abortus* Δ*flgJ* (pBV1-*flgJ*)-*gfp* strains with EEA1 protein **(A)**, LAMP1 protein **(B)**, and calnexin protein **(C)**. Results were expressed as mean ± standard deviation (SD), **P < 0.01 significant.

To study the effect of the elimination of the *flgJ* gene in intracellular traffic, the protection test with gentamicin was used (Starr et al., 2008), infecting macrophages with *B. abortus* 2308, *B. abortus* Δ*flgJ* or the complemented strain. No significant differences were seen at 15 min p.i. between, *B. abortus* 2308 and *B. abortus* Δ*flgJ* (*P* > 0.05), since they both showed similar percentages of co-localization with the early endosome marker EEA1 (39% and 42% for *B. abortus* 2308 and *B. abortus* Δ*flgJ*, respectively) (Figure 5A). Then, at 4 h p.i. no significant difference in co-localization with late endosome marker LAMP1 was found between *B. abortus* 2308 (23%) and *B. abortus* Δ*flgJ* (11%) (*P* > 0.05) (Figure 5B). Interestingly, in contrast to the wild-type strain, *B. abortus* Δ*flgJ* did not significantly co-localize with the ER marker calnexin protein at 12 h p.i. (*P* < 0.05) (Figure 5C). No difference in intracellular trafficking was observed between the wild-type and *B. abortus* Δ*flgJ* (pBV1-*flgJ*). These data suggest that the *flgJ* mutant was prevented from reaching the ER, which is fundamental to intracellular survival of *B. abortus* 2308.

### Survival of *B. abortus* 2308 Δ*flgJ* in Mice

The effect of the *flgJ* gene deletion on bacterial virulence was determined, analyzing the bacterial loads present in the spleens of infected mice. We found that the survival of *B. abortus* 2308 Δ*flgJ* in mouse spleen was significantly reduced after the 4 weeks p.i. from 6.28 ± 0.39 log_10_ CFU/spleen in the second week p.i. to 3.22 ± 0.07 log_10_ CFU/spleen in the fourth week p.i. (*P* < 0.05). Compared to the wild-type strain, the reduction was 1.22 log_10_ CFU/spleen units at 4 weeks p.i. (Table 3). On the other hand, there were significant differences in the persistence of the *B. abortus* 2308 Δ*flgJ* mutant and the complemented strain at 4 weeks (Table 3). These are compelling data that suggest that the presence of the FlgJ protein is important for bacterial persistence in BALB/c mice.

**Table 3:** Spleen colonization of BALB/c mice with *B. abortus* wild-type 2308, *B. abortus* 2308 Δ*flgJ* mutant, and complemented *B. abortus* 2308Δ*flgJ* (pVB1-*flgJ*) strains.

***B. abortus* strains**	**Two-weeks post infection**	**Four-weeks post infection**
*B. abortus* 2308	5.95 ± 0.16	4.44 ± 0.56
*B. abortus* 2308 Δ*flgJ*	6.28 ± 0.39	3.22 ± 0.07^*^
*B. abortus* 2308 Δ*flgJ* (pVB1-*flgJ*)	6.24 ± 0.51	3.80 ± 0.76

*Log_10_ CFU of B. abortus strains in the spleens of BALB/c mice infected intraperitoneally with 5 x 10^4^ CFU wild-type B. abortus 2308, B. abortus 2308ΔflgJ mutant or complemented B. abortus 2308ΔflgJ (pVB1-flgJ) strains. Statistical analyses were made comparing mutant with wild type and complemented strains*.

* *P < 0.05 showing a number significantly less of CFU/spleen compared to B. abortus 2308*.

## Discussion

The flagellum is an important virulence factor used for motility of various pathogenic bacteria to reach their specific infection site, avoiding hostile environments and accessing nutrients (Moens and Vanderleyden, 1996). It has been demonstrated that the flagellar system is essential for the infectious cycle and persistence of *Brucella* in mammalian hosts (Fretin et al., 2005; Zygmunt et al., 2006; Al Dahouk et al., 2017). A polar flagellum has been identified in *B. melitensis*, which is expressed under very specific infection conditions (Briones et al., 2001). The importance of this macrostructure in the physiology or pathogenicity of *Brucella* species has not been described; However, *B. melitensis* mutant strains for some flagellar proteins such as *fliF, flhA, motB*, or *flgE* are less able to survive intracellularly in mouse spleen than the parental strain (Fretin et al., 2005). Although *B. abortus* contains all the genes for a functional flagellum, it has not yet been described yet. However, this bacterium possesses a flagellar-peptidoglycan hydrolase FlgJ in the GI-3, which could be involved in the virulence of this species (Li et al., 2012). Based on this information, in this study the open reading frame BAB1_0260, encoding for the flagellar FlgJ protein, from *B. abortus* 2308 was deleted to evaluate if its product is involved in the virulence of this bacterium (Ratushna et al., 2006; He and Xiang, 2010; Zhang et al., 2012).

Here we demonstrated that the deletion of FlgJ, a protein involved in the flagellum assembly and PG-remodeling, was not lethal for the *B. abortus* strain. This protein has a N-terminal half with function scaffold or cap essential for flagellar rod assembly and a C-terminal half with peptidoglycan (PG)-hydrolyzing activity that facilitates the rod penetration into the PG (Nambu et al., 1999; Hirano et al., 2001). This hydrolase function makes it possible to remodel the PG and it facilitates the passage of the flagellum (rod) through of the β-N-acetylglucosamine and β-N-acetylmuramic acid sugars (Hirano et al., 2001; Zaloba et al., 2016). Furthermore, it has been reported that bacteria mutants for *flgJ* fail to produce periplasmic and external flagellar components such as the rods, hooks (FlgE) or filaments (FlaB, in *B. abortus* is called FliC) proteins due to their peptidoglycan hydrolase activity, which is necessary to penetrate the PG layer during flagellar formation, but it is also required for the cell growth and division (Vollmer et al., 2008; Zhang et al., 2012). Interestingly, a comparative analysis shows that, although *B. abortus* FlgJ differs in the length of amino acid sequences, it contains the same domains involved in the rod assembly and in the PG-hydrolyzing activity (glucosaminidase and LYZ2) as the flagellar FlgJ protein of *E. coli* (Marchler-Bauer et al., 2017).

Considering that *B. abortus* is a “non-mobile” bacterium, the hydrolyzing activity of FlgJ over the PG could play an important role in the growth of this bacterium, specifically during the remodeling of the PG during its cell division, which would explain why deletion of the *flgJ* gene significantly reduced its growth, which was observed by a decreased ability to adapt to the lag phase and at the beginning of the exponential phase. Furthermore, although the deletion of the *flgJ* gene showed an important role in the adherence to the polystyrene surface, which would demonstrate that FlgJ participates in the adherence or in the secretion of proteins involved in the process, is highly probable that the biomass adhering of *B. abortus* 2308 *flgJ* is directly correlated to the levels of bacterial growth and division. A reduced bacterial adherence would affect negatively the colonization of the host‘s tissues, where several factors participate, including the flagellum (Klemm et al., 2010). In this process, several bacteria produce biofilm, a structure that has not been described in *B. abortus*, but which is associated with the same Quorum Sensing signals (VjbR) involved in the expression of the flagellar genes in *B. melitensis* (Taminiau et al., 2002; Delrue et al., 2005). Finally, deletion of *flgJ* gene in E. coli K-12, a flagellated bacterium, abolished its movement. Nevertheless, it was slightly restored by the complementation with the *B. abortus* flgJ gene. This may have occurred due to a possible structural and functional similarity between these two phylogenic distant proteins, which was supported by slight growth *in vitro* of *E. coli flgJ* complemented strain. Therefore, the deletion of the *flgJ* reduces the biomass adhering and motility, however, the flagellar production and motility are downregulated in the biofilm cells (Rossi et al., 2018). These observations would support the idea that the biomass adhering of *B. abortus* 2308 *flgJ* is associated to bacterial growth and division, being evident that FlgJ would participate in the normal life cycle of *B. abortus* 2308.

The effect of FlgJ in the physiology of the *B. abortus* mutant strain for *flgJ* gene was affected, reducing significantly intracellular survival in professional and non-professional phagocytic cells. This ability of *B. abortus* is similar to that described for mutant strains whose genes are encoded in genomic island 3 of *B. abortus* (GI-3), a genomic segment the FlgJ protein is encoded (Céspedes et al., 2011; Ortiz-Román et al., 2014). In addition, in epithelial cells and macrophages *B. abortus* mutant for the FlgJ protein was unable to reach its replicative niche, which is associated with the endoplasmic reticulum (de Bagues Maria-Pilar et al., 2005). In the first instance, this leads us to think that FlgJ is a virulence factor whose deletion in this mutant strain makes it more susceptible to be carried toward the lysosomal compartments and its subsequent degradation. This lysosomal degradation pathway has also been reported in several *Brucella* mutants for important virulence factors such as, cyclic β-1,2-glucans (CβGs), LPS or virB genes codifying of the type IV secretion system (Celli et al., 2003; Haag et al., 2010; Gomes et al., 2013). This subcellular compartment (ER) was achieved by *B. abortus* wild type at 12 p.i.; however, the *B. abortus* mutant for FlgJ simultaneously showed a reduced capacity to replicates in this compartment of the host's eukaryotic cells. consequently, a high number of bacteria is degraded by the macrophages. This is how the deletion of FlgJ affects the fitness of *B. abortus*. Based on our results, we believe that FlgJ hydrolase activity is required for the basic physiology of this bacterium, likely during the cellular infection process, which negatively affect the capacity of *B. abortus* to escape from the endosomal/phagolysosomal pathway and develop a replicative niche in macrophages, which allows it to survive and replicate intracellularly in eukaryotic cells.

The cellular infection models were positively correlated with the infection of mice, where the mutant *B. abortus* 2308 Δ*flgJ* strain, compared with *B. abortus* 2308 and *B. abortus* ΔflgJ (pBV1-flgJ) complemented strain, was significantly eliminated from spleen of mice at 4 weeks p.i. because mutant strains for *FlgJ* reduces the expression of FlaB (FliC in *Brucella*), a protein of flagellum filament in *Borrelia burgdorferi* (Zhang et al., 2012), it would be expected that periplasmic or filament FliC protein of *Brucella*, would not be expressed during the infection of mice. In *B. melitensis*, the expression and recognizing of FliC protein determine the activation of the innate immune response and modulates the systemic persistence of *Brucella* during the infection (Terwagne et al., 2013). Besides, differences between *in vivo* and *in vitro* assays were observed, where *B. abortus* was remain for longer period of time in mice than macrophages or epithelial cells. These results would depend of innate immune sensors for flagellar protein, where FliC is targeted for the cytosolic receptor NLRC4 during infection of mice; while, alternative pathways could recognize this protein in macrophages cultured *in vitro* (Terwagne et al., 2013). Furthermore, it has been reported that *in vivo* infections can be more persistent than *in vitro*, because *B. abortus* establishes a persistent infection in a “protected niche” (e.g., B cells) that obstructed an effective immune response within the host or by the formation of granulomas where bacteria persisted in spleen (Goenka et al., 2012; Grilló et al., 2012). Furthermore, at 4 weeks p.i. the complemented strain had a lower response in comparison than wild type, but higher than the mutant strain, a tendency to lose its capacity for infection and persistence in the host as well as *B. abortus* Δ*flgJ*. This may be because the mice were not treated with ampicillin, an antibiotic required for maintenance of plasmid complementation, which was not used because it could not reach the concentration required at the infection site, cross the host's cell membranes or because the mice treated were able to change their natural microbiota, thereby altering the results (Krute et al., 2016). Consequently, these results demonstrate that FlgJ is an important protein in the virulence of this pathogen and that this flagellar protein of *B. abortus* is similar to the results reported for several mutants for flagella structural proteins such as the MS ring (FliF), the P ring (FlgI) and the filament (FliC) during the infection process of mice (Fretin et al., 2005).

Therefore, the results demonstrate that the flgJ gene product (FlgJ protein) significantly contributed to the virulence of *B. abortus* strain 2308, supporting the hypothesis that the absence of the FlgJ flagellar protein affects intracellular survival of *B. abortus* 2308 and the establishment of a systemic infection in a murine model. Therefore, assuming that this protein acts in the assembly of a polar flagellum, several questions arise regarding its functions during infection or perhaps in the secretion of proteins such as a type 3 secretion system (T3SS), which has not been described in these bacteria (Ratushna et al., 2006). Based on the results described here, we can demonstrate that *flgJ*, a gene encoded by the ORF BAB1_0260 conserved in the GI-3, contributes to the fitness of *B. abortus*, which significantly reduced its virulence in *in vitro* and *in vivo* experimental models.

## Conclusion

With these results it can be pointed out that FlgJ (BAB1_0260) is a protein that participates in many important processes in *Brucella abortus* 2308 infection events and in establishing of a replicative niche, mobility and biomass production, which are essential to colonization of the host cell.

## Data Availability Statement

The datasets generated for this study are available on request to the corresponding author.

## Ethics Statement

The animal study was reviewed and approved by Institutional Bioethics Committee of the Universidad de Concepción and the Bioethics and Security Committee of the Faculty of Biological Sciences in the Universidad de Concepción.

## Author Contributions

RC-R writing and discussion of the result, survivor intracellular experiment, evaluation of biofilms. LG bacterial colonization assay and statistical analysis of the results. FA study of intracellular traffic by confocal microscopy. WS construction of mutants. FC construction of recombinant plasmid and evaluation of motility assay. SC bacterial colonization assay. RV review and proposal of conclusions. AO programming and monitoring the experiment, performing the analysis and discussion, writing the manuscript and principal investigator at the FONDECYT grant that funded this work.

## Conflict of Interest

The authors declare that the research was conducted in the absence of any commercial or financial relationships that could be construed as a potential conflict of interest.
